# Draft genome sequences of six bacteria isolated from polylactic acid samples from the Mediterranean Sea

**DOI:** 10.1128/MRA.00794-23

**Published:** 2023-10-19

**Authors:** Jorge Rojas-Vargas, Libertad Adaya, Ana Luzia Lacerda, Maria-Luiza Pedrotti, Alejandro Sanchez-Flores, Liliana Pardo-López

**Affiliations:** 1 Departamento de Microbiología Molecular, Instituto de Biotecnología, UNAM, Col. Chamilpa, Cuernavaca, Morelos, México; 2 Laboratoire d’Océanographie de Villefranche sur mer (LOV), UPMC Université Paris , CNRS UMR, Sorbonne Université, Villefranche sur Mer, France; 3 Unidad Universitaria de Secuenciación Masiva y Bioinformática, Instituto de Biotecnología, Universidad Nacional Autónoma de México, Cuernavaca, México; California State University San Marcos, San Marcos, California, USA

**Keywords:** marine bacteria, plastic degradation, Mediterranean Sea

## Abstract

Here, we report the draft genome sequences of six marine strains isolated from plastic samples incubated in the Mediterranean Sea. Genomic analyses place these strains within the *Alkalihalobacillus*, *Bacillus*, *Halomonas*, and *Marinobacter* genera. Examining the genomes of these non-typical environmental bacteria increases our comprehension of microorganism biology and their potential uses.

## ANNOUNCEMENT

In this study, six strains were isolated from 1 cm × 1 cm polylactic acid plastic squares submerged in stainless steel cages incubated 5 m deep in the Mediterranean Sea (43°41'59.71"N, 7°18'59.94"E) for 3 months. These plastic samples were retrieved from the sea and introduced into a marine broth (MB) medium, followed by incubation at 30°C at 200 rpm for 5 days. Once the population capable of colonizing plastics was reactivated, culture media were collected and inoculated into ONR7 basal medium supplemented with 0.3% of two plastic resins, polyurethane (Polycrylic, Sherwin Williams, USA), and polyethylene (Wyn de Mexico), respectively. These cultures were incubated at 30°C at 200 rpm for 10 days. For bacterial isolation, serial dilutions of the cultures containing plastic resins were prepared and subsequently plated on ONR7 medium agar plates supplemented with the same resins. Colonies displaying a halo of degradation were selected. The isolates were cultivated individually in MB medium overnight, followed by genomic DNA extraction using the Quick-gDNA Miniprep Kit (Zymo Research), according to the manufacturer’s specifications. Illumina MiSeq platform was used for sequencing at the massive sequencing unit of the Institute of Biotechnology (IBt-UNAM, Cuernavaca). Illumina sequencing library was prepared using the Illumina DNA Prep Library Kit (Illumina, Inc.), where the DNA was enzymatically fragmented. Sequencing statistics are described in Table 1. Read quality and trimming were performed with FastQC software v.0.11.9 (1) and Trimmomatic v0.39 (2), respectively. Assembly was done with SPAdes v3.13.0 (3) with the option --careful. Assembly metrics were assessed with QUAST v5.0.2 (4) and the completeness and contamination with CheckM v1.2.2 (5). Gene prediction and functional annotation were performed with the NCBI Prokaryotic Genome Annotation Pipeline (6). Default parameters were used for all software unless otherwise noted. The obtained assemblies had sequencing depths between 219 and 295×, and the G + C contents and genome sizes were found to be 39.5% to 58.0% and 3.45 Mbp to 4.57 Mbp, respectively (Table 1).

**TABLE 1:** Genomic features and accession numbers for the six Mediterranean strains

Genome features	*Marinobacter salarius* MDS1	*Marinobacter* sp. MDS2	*Halomonas meridiana* MDS3	*Bacillus halotolerans* MDS4	*Alkalihalobacillus macyae* MDS5	*Bacillus safensis* MDS6
Assembly size (bp)	4,572,548	3,449,993	3,718,152	4,094,543	4,356,540	3,686,322
Sequencing depth (×)	219	219	295	279	268	286
G + C content (%)	56.5	55.0	58.0	43.5	39.5	41.5
No. of paired reads (2 × 301 bp)	1,662,955	1,255,964	1,821,641	1,900,892	1,945,089	1,748,462
No. of scaffolds	18	10	26	29	31	9
N50 (bp)	1,087,040	2,302,689	327,719	330,502	237,567	1,922,558
L50	2	1	4	4	7	1
Completeness	99.61	98.07	99.52	99.41	99.35	99.22
Contamination	1.07	1.74	0.19	0	0.65	0.29
No. of genes	4,148	3,186	3,470	4,230	4,479	3,787
No. of CDSs	4,091	3,134	3,377	3,996	4,381	3,708
No. of tRNAs	47	43	57	69	79	59
No. of rRNAs	5	5	7	18	14	15
GenBank accession no.	GCA_030718145.1	GCA_030718085.1	GCA_030718065.1	GCA_030718045.1	GCA_030718105.1	GCA_030718025.1
SRA accession no.	SRR25519351	SRR25519350	SRR25519349	SRR25519348	SRR25519347	SRR25519346

For taxonomic identification, average nucleotide identity (ANI) values were calculated using PyANI v0.2.12 (https://github.com/widdowquinn/pyani) against the three closest genomes for each strain determined by GTDBtk v2.1.0 (7) (see Figure 1 posted at https://figshare.com/articles/figure/Supplementary_Figure_PyANI_Mediterranean/23827053). The assemblies were retrieved from the NCBI portal (https://www.ncbi.nlm.nih.gov/assembly consulted on 6 June 2023). The highest ANI values suggest that the strain MDS1 is a *Marinobacter salarius* (98.72% compared to *M. salarius* SMR5), MDS2 is a novel *Marinobacter* species (89.40% compared to *M. litoralis* CARE-V18), MDS3 is an *Halomonas meridiana* (98.03% compared to *H. meridiana* SCSIO_43005), MDS4 is a *Bacillus halotolerans* (98.10% compared to *B. halotolerans* ATCC 2509), MDS5 is an *Alkalihalobacillus macyae* (96.95% compared to *A. macyae* DSM16346), and MDS6 is a *B. safensis* (99.00% compared to *B. safensis* FO-36b).

Secondary metabolite-related gene clusters were predicted using antiSMASH v7.0 in strict mode (8), revealing their presence in all strains. Each genome has at least one betalactone gene cluster, and about half possess for terpenes, NRPS, ectoine, redox factor, T3-PKS, siderophores, and metallophores (see Figure 2 posted at https://figshare.com/articles/figure/Supplementary_Figure_PyANI_Mediterranean/23827053).

## Data Availability

The whole-genome sequence data and raw sequences are available at NCBI under the Bioproject accession number PRJNA1001137.

## AFTER EPUB

*[This article was published on 19 October 2023 with two misnamed figures cited without a link to the website at which they can be found. The figure citations were updated in the current version, posted on 31 October 2023.]*

## References

[1] Andrews S . 2010. FastQC: a quality control tool for high throughput sequence data. Available from: http://www.bioinformatics.babraham.ac.uk/projects/fastqc

[2] Bolger AM , Lohse M , Usadel B . 2014. Trimmomatic: a flexible trimmer for illumina sequence data. Bioinformatics 30:2114–2120. doi:10.1093/bioinformatics/btu170 24695404 PMC4103590

[3] Bankevich A , Nurk S , Antipov D , Gurevich AA , Dvorkin M , Kulikov AS , Lesin VM , Nikolenko SI , Pham S , Prjibelski AD , Pyshkin AV , Sirotkin AV , Vyahhi N , Tesler G , Alekseyev MA , Pevzner PA . 2012. SPAdes: a new genome assembly algorithm and its applications to single-cell sequencing. J Comput Biol 19:455–477. doi:10.1089/cmb.2012.0021 22506599 PMC3342519

[4] Gurevich A , Saveliev V , Vyahhi N , Tesler G . 2013. QUAST: quality assessment tool for genome assemblies. Bioinformatics 29:1072–1075. doi:10.1093/bioinformatics/btt086 23422339 PMC3624806

[5] Parks DH , Imelfort M , Skennerton CT , Hugenholtz P , Tyson GW . 2015. CheckM: assessing the quality of microbial genomes recovered from isolates, single cells, and metagenomes. Genome Res 25:1043–1055. doi:10.1101/gr.186072.114 25977477 PMC4484387

[6] Li W , O’Neill KR , Haft DH , DiCuccio M , Chetvernin V , Badretdin A , Coulouris G , Chitsaz F , Derbyshire MK , Durkin AS , Gonzales NR , Gwadz M , Lanczycki CJ , Song JS , Thanki N , Wang J , Yamashita RA , Yang M , Zheng C , Marchler-Bauer A , Thibaud-Nissen F . 2021. RefSeq: expanding the prokaryotic genome annotation pipeline reach with protein family model curation. Nucleic Acids Res 49:D1020–D1028. doi:10.1093/nar/gkaa1105 33270901 PMC7779008

[7] Chaumeil P-A , Mussig AJ , Hugenholtz P , Parks DH . 2019. GTDB-TK: a toolkit to classify genomes with the genome taxonomy database. Bioinformatics 36:1925–1927. doi:10.1093/bioinformatics/btz848 31730192 PMC7703759

[8] Blin K , Shaw S , Augustijn HE , Reitz ZL , Biermann F , Alanjary M , Fetter A , Terlouw BR , Metcalf WW , Helfrich EJN , van Wezel GP , Medema MH , Weber T . 2023. antiSMASH 7.0: new and improved predictions for detection, regulation, chemical structures and visualisation. Nucleic Acids Res 51:W46–W50. doi:10.1093/nar/gkad344 37140036 PMC10320115

